# Biogenic Selenium Nanoparticles: Potential Solution to Oxidative Stress Mediated Inflammation in Rheumatoid Arthritis and Associated Complications

**DOI:** 10.3390/nano11082005

**Published:** 2021-08-05

**Authors:** Ayesha Rehman, Peter John, Attya Bhatti

**Affiliations:** Department of Healthcare Biotechnology, Atta-ur-Rahman School of Applied Biosciences, National University of Sciences and Technology, Islamabad 44000, Pakistan; ayesharehman.iiui@gmail.com (A.R.); attyabhatti@gmail.com (A.B.)

**Keywords:** selenium, nanoparticles, biological methods, inflammation, oxidative stress, rheumatoid arthritis

## Abstract

Rheumatoid arthritis (RA) is a common chronic inflammation-mediated disorder having systematic complications. RA triggers a self-directed inflammatory and immunological cascade that culminates in joint destruction. Though a range of treatment options are available, none of them are without adverse effects and this has led researchers to search for alternative solutions. Nanomedicine has emerged as a powerful therapeutic alternative, and selenium (Se) is an essential micronutrient trace element that has a crucial role in human health and disease. Selenium nanoparticles (SeNPs) derived from biological sources, such as plants, bacteria, fungi, and proteins, have exhibited remarkable candidate properties and toxicological profiles, and hence have shown potential to be used as antirheumatic agents. The potential of SeNPs can be attributed to the effect of functional groups bound to them, concentration, and most importantly to their nano range size. The antirheumatic effect of SeNPs is considerable due to its potential in amelioration of oxidative stress-mediated inflammation via downregulation of radical and nonradical species, markers of inflammation, and upregulation of inherent antioxidant defenses. The size and concentration impact of SeNPs has been shown in the subsequent antioxidant and anti-inflammatory properties. Moreover, the article emphasizes the role of these biogenic SeNPs as a notable option in the nanomedicine arena that needs to be further studied as a prospective remedial alternative to cure RA and medication-related adverse events.

## 1. Introduction

Rheumatoid arthritis (RA) is a common chronic inflammation-mediated disorder [1]. It is a long-lasting condition described as inflammation of diarthrodial joints leading to symmetrical polyarthritis and synovial hyperplasia (swelling) that results in progressive destruction of cartilage and bones and loss of articular function that leads to the eventual deformation of joints [2]. Moreover, it is a systematic autoimmune disorder that can alter multiple organ systems [3]. The inflammation implicated also affects the brain (decreased cognitive abilities and fatigue), lungs (fibrotic disorders), liver (increased response of acute phase proteins and chronic lack of red blood cells), exocrine glands (secondary Sjogren’s syndrome), muscles (sarcopenia), as well as bones (osteoporosis) [4]. RA has a global prevalence between 0.5% and 1% alongside a greater incidence in females as compared to males and is ranked next to disorders like osteoarthritis and gout as a foremost reason of disability [5,6,7]. Depending upon the degree of the disease, age of onset, and related comorbidities, life expectancy is 3–10 years lower in RA patients than that of the general population [8]. The associated death rate is twice as high in affected people than in the normal population and this lag appears to be increasing [9].

The exact etiological milieu for RA is still not certain, but as an example of a chronic inflammation-mediated autoimmune disorder, it has been correlated to oxidative stress (OS), a state wherein a pool of reactive oxidative species (ROS) upregulates actively, either due to their enhanced generation, the decline in antioxidative defense mechanisms, or the combined effects of both, thus leading to altered redox signaling that is involved in the maintenance and progression of the disorder [10,11]. The therapeutic options for patients suffering from RA include nonsteroidal anti-inflammatory drugs (NSAIDs), glucocorticoids (GC), and disease modifying antirheumatic drugs (DMARDs), but all of these available therapeutic remedies have associated adverse effects [1]. Thus, there is a prominent need to develop and test novel drugs that intend to ameliorate inflamed synovial joints and mitigate bone damage. Selenium (Se) is an essential micronutrient trace element having a crucial role in normal human functioning and has prominent relevance to several pathophysiological conditions [2].

In this regard, one of the most promising therapeutic solutions for RA is ‘nanomedicine’ [3] and has captured quite the amount of attention. Selenium nanoparticles (SeNPs) have become the centerpiece of attention due to their exclusive physical and chemical properties [4]. The SeNPs play an essential role in the antioxidant defense system that is crucial for reduction of oxidative stress [5]. The focus of the present review is to report the recent updates that highlight the antioxidant and anti-inflammatory potential of biogenic SeNPs as a significant and possible therapeutic option to medicate RA patients.

## 2. Natural Dietary Selenium

Se is an essential micronutrient trace element having a crucial role in normal human functioning and prominent relevance to several pathophysiological conditions [2]. Inorganic Se can be found in four oxidation states, i.e., selenite (Se^+4^), selenate (Se^+6^), elemental Se (Se^0^), and selenide (Se^−2^), and can be converted into organic and bioavailable forms, e.g., selenocysteine (Sec) and selenomethionine (SeMet), through biological processes [6]. Sec is the 21st amino acid and SeMet is a Se conjugated natural amino acid also known as the most suitable form for nutritional supplementation of Se due to its abundant bioavailability [7]. Sec is present at the active sites of the enzymes as a cofactor and is therefore important for their catalytic activity [6]. In mammals, 30 selenoproteins have been recognized and 25 selenoproteins have been proven to be found in humans [8]. The expression of these eukaryotic selenoproteins is specific to respective tissues depending on the availability of Se and can be regulated through hormones [2,6,9,10].

### Selenium Absorption and Bioavailability

**Figure 1:**
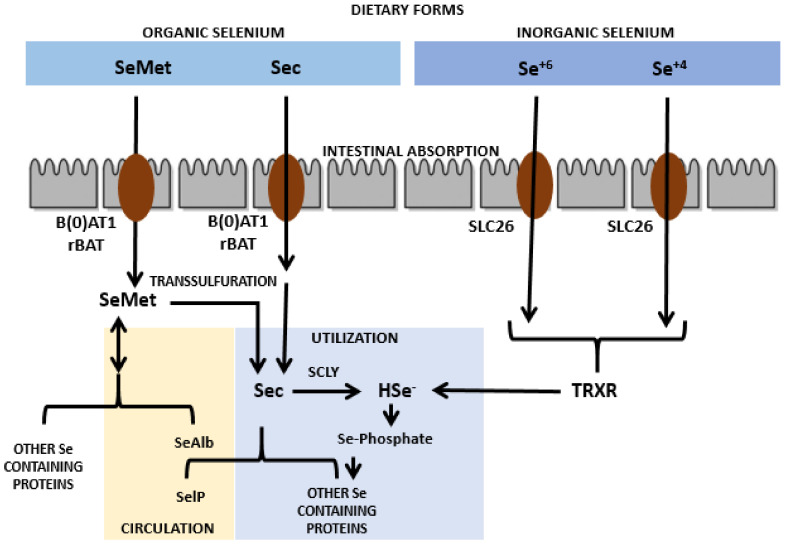
Absorption and metabolism of selenium nanoparticles.

Sec is present in animal proteins and SeMet in plant proteins [11]. Organic as well as inorganic Se (except Se^+4^) has an absorption rate of about 80% upon intake in normal physiological events taking place in proximal jejuna and duodena [12]. Though the mechanism of Se absorption is still not clear, Sec and SeMet are shown to be transported via amino acid transporters in the intestine, especially by the sodium-dependent neutral amino acid transporter B(0)AT1 as well as by the neutral and basic amino acid transport protein rBAT, respectively. In contrast, Se^+6^ has been revealed to be absorbed via the solute carrier 26 (SLC26) family of multifunctional anion exchangers [13,14,15]. The intracellular transport of Se is conceivably analogous to that of amino acids [15]. Upon absorption, Sec is transported intact or via a mechanism that is still unknown, however, SeMet is transported as Se albumin (SeAlb) to the liver via blood circulation [13]. Hepatocytes are able to add SeMet to replace methionine in proteins and the leftover SeMet is transformed into Sec by the pathway of trans-sulfuration in the liver as well as in other tissues. The resultant and diet-derived Sec is then transformed into Se^−2^ via selenocysteine β-lyase (SCLY). In contrast, Se^+4^ can be broken down to Se^−2^ via thioredoxin reductases (TrxRs) in the liver and can also be converted into Se^−2^ by the glutathione glutaredoxin pathway [14]. Figure 1 shows the graphical depiction of the absorption and metabolism of Se. The resultant Se^−2^ is further used to form Sec tRNA that is required for the synthesis of the selenoproteins needed by the human body [14,16]. The chemical form of Se in humans has an influence on the rate of absorption in the human body. Sec, SeMet, and Se^+6^ have an absorption rate of about 70 to 90%, however for Se^+4^ it has been observed to not exceed 60%. Overall, it has been shown that the absorption of this trace nutrient is better in its organic form taken via a diet rich in protein [12,13]. The absorption of this nutrient is also facilitated when it is taken with sources that are rich in vitamins A, D, and E [17]. However, the absorption of SeMet and Sec is lowered if there is more intake of their sulfur analogs (methionine and cysteine) due to the structural resemblances with these amino acids [18,19]. Moreover, there are some medicines that interfere with sufficient Se absorption such as chelators, for instance, deferiprone. Deferiprone is shown to eliminate extra iron in patients of thalassemia that have undergone blood transfusions, but several reports have indicated a change in serum Se amounts that may be a result of the administration of deferiprone [20,21]. Of interest, this disease can increase the frequency of RA appearance. This might be a result of the deposition of iron in the synovial tissues or because of iron chelators due to the generation of ROS in iron exchange [22].

## 3. Serum Selenium Status in Rheumatoid Arthritis

In the past, lowered serum concentration of trace micronutrients has been demonstrated as a frequent event in autoimmune diseases [23]. Epidemiological reports proved that a low Se status can be a risk factor for RA, indicating the significance of antioxidants in controlling the maintenance and progression of the disease [24,25,26,27,28,29,30,31,32,33]. Some other recent studies have also supported the relevance of Se status in RA [34,35,36]. Moreover, Na Yu et al. also reported the relation between serum Se levels and RA through a meta-analysis approach. In this study, they reported a meta-analysis from 14 case control studies that included 716 participants and showed significant association between RA and low serum Se concentration [37]. Given that, it has been reported since long that observed serum Se amount is less in RA patients than in controls [38]. Francisco et al. also discussed the relevance of Se status in detail in context to RA [39]. Nonetheless, though it is not clear if Se deficiency is a cause or outcome of the incident of the disorder, the decreased levels can result in the progression of the disease since they have been linked to the creation of ROS and related inflammatory states [40].

### Selenium Supplementation in RA

It has been reported that Se supplementation improves the condition of patients as well as reduces inflammation levels in experimental models, such as the granuloma pouch exudate, and in lupus mice or in the adjuvant arthritis in rats [41]. Evidence has suggested that Se can decrease inflammation in autoimmune disorders [42]. One report revealed that Se supplementation has an antioxidant effect as it upregulates selenoproteins and downregulates inflammation in autoimmune disorders [43]. The significance of Se supplementation, however, is still not distinct for the treatment of RA patients based on confusing data [44,45]. Moreover, the associated toxic effect of Se for the treatment of arthritis as an antioxidant supplement is debatable [46]. The foremost drawback regarding the intake of Se as a supplement is its toxicity and bioavailability [42]. Goldhaber et al. reported that selenosis occurs due to acute/chronic intake of excess Se, characterized by fingernail changes and brittleness, skin rashes, hair loss, garlic breath, gastrointestinal disturbances, and abnormal functioning of the nervous system. Other related harmful effects include abnormal production of thyroid hormones and growth hormones as well as an insulin-like growth factor metabolism leading to disrupted endocrine functions [47].

## 4. Current RA Medication

Conventional treatment options for RA patients comprise of NSAIDs, GC, conventional synthetic disease-modifying anti-rheumatic drugs (csDMARDs), and biologic DMARDs (bDMARDs), and all these available therapeutic remedies have associated side effects. Table 1 summarizes the diverse treatment options available for RA and their associated side effects.

NSAIDs have an analgesic effect in the initial phase of RA, but because of their inadequate usefulness, failure to control long-term disease progression, and numerous adverse effects such as cardiovascular risk, gastro-intestinal disorders, and renal malfunction, their usage is linked to multiple complications [48,49]. GCs act as anti-inflammatory mediators and can be prescribed to RA patients during the first two years of treatment, however, their systemic use is discouraged due to the associated adverse effects of long-term application, including cardiovascular disorders, osteoporosis, impaired glucose metabolism (resistance to insulin), skin thinning, hypertension, diminished lesion repair, and obesity [48,50]. Conventional synthetic DMARDs have specific anti-rheumatic potential but can bring about serious adverse reactions such as interstitial pneumonitis, myelosuppression, hepatic cirrhosis, retinopathies, hypersensitivity, and allergic reactions [51]. Biologic DMARDs have had notable results, but multiple complications are linked to their use, including risk of acute bacterial infections, failure to keep up response over time, and high cost [52]. Lately, the breakthrough of Janus kinase (JAK) inhibitors led to the introduction of a novel class of drugs to cure RA, known as targeted synthetic DMARDs (tsDMARDs) [53]. However, these drugs, too, have related adverse effects [54,55,56,57].

## 5. Nanomedicine as a Potential Solution

RA continues to be a challenging disorder because all the above mentioned recommended therapies do not often lead to a cure and are linked to frequent drug resistance and related side effects [58]. Hence, it is crucial to develop and test novel drugs that target inflamed joints and mitigate damage. In this regard, one of the most promising therapeutic solution for RA is nanomedicine [3]. Hundreds of diverse nanomedicinal formulations have been prepared and assessed over the years for various kinds of maladies. However, about 50 of such formulations are at present approved for clinical usage and several nanomedicines are going through trials [59]. Nanoparticles (NPs) are defined as nano-range submicroscopic particles that have unique properties such as large surface area, nano size, surface charge, and chemistry, solubility and multifunctionality [60]. NPs are deemed as being in a transitional stage between individual molecules and the analogous bulk materials, which allows them to possess peculiar properties that are unique from their molecular and bulk analogue counterparts [60,61]. Based on their unique properties, nanoscale materials and devices can interact with biomolecules from both the inside and on the cell surface that have the potential to detect disorders and deliver treatments. Hence, NPs have revolutionized healthcare as they facilitate research and development, help with early detection, enhance molecular imaging, and enable prevention, diagnosis, and control [62]. Improved permeability and enhanced retention (EPR) is one of the most significant criteria for the cure of RA and other chronic inflammatory diseases due to the extensive systemic nature of inflammation [63]. Nanomedicine can actively or passively build up in the inflamed joints through EPR over time [64]. Drugs loaded in nanocarriers, combined with the pathophysiological features of inflamed joints, can enhance the bioactivity and bioavailability of remedial therapeutics and might promote the specific targeting of inflamed joints [3]. NPs having antioxidant properties have been implicated as a plausible approach in the treatment of OS-mediated inflammatory disorders such as inflammatory bowel disease (IBD), neurodegenerative diseases, cardiovascular diseases (CVDs), asthma, diabetes, and arthritis [65,66,67]. The most studied NPs for the treatment of RA are dendrimer, liposome, polymeric, and metallic NPs [68].

## 6. Selenium Nanoparticles

SeNPs have received attention due to their exclusive physical and chemical properties (i.e., mechanical, electrical, catalytic, and opt-magnetic properties) that are exhibited when this element is scaled down to the nano range as a result of high spatial confinement of nanomaterials, high surface-to-volume ratio, and large surface energy [69,70,71]. SeNPs, due to remarkable photoreactive, biocidal, anticancer, antidiabetic, antioxidant, antimicrobial, and anti-inflammatory properties in the healthcare arena, are being used in antimicrobial coatings, diagnostics, medical devices, nutritional supplements, and nanotherapeutics [72,73]. SeNPs have significantly emerged as dual targeting modality with both pro-oxidant and antioxidant potential dependent on subsequent duration, dose, and frequency as well as oxidation state. The pro-oxidant potential of SeNPs has been exploited in anticancer agents (chemotherapeutic drugs carriers). These NPs fundamentally localize in the malignant cells and lead to the production of reactive oxygen species, and hence cause cytotoxicity. The pro-oxidant mechanism of SeNPs follows the reduction of nanoselenium via thioredoxin- and glutaredoxin mediated redox signaling that leads to the generation of Se^2−^ anion through the consumption of NADPH+H^+^ and stimulated production of ROS [74]. SeNPs play an important role in the antioxidant defense system, which is essential for reducing oxidative stress [5]. Se is an integral part of selenoproteins, such as glutathione peroxidases (GPxs) and TrxRs, which are needed for several biochemical reactions involved in normal antioxidant defenses [75]. SeNPs have been studied in different inflammation and redox imbalance-mediated disorders, such as cancer, diabetes, nephritis, and arthritis, and showed potential remedial uses [72].

### 6.1. Pharmacokinetics and Toxicological Profile of SeNPs

Pharmacokinetic parameters, such as absorption, distribution, metabolism, and excretion, as well as toxicological profiles have an important role in selecting a good candidate for therapeutic roles. Oral intake of NPs is regarded as the most suitable and cost-effective mode of supplementation. Nonetheless, the absorption of NPs is hindered by two gastrointestinal barriers: the intestinal mucosa and the mucus covering the intestinal mucosa [76]. In theoretical terms, NPs can pass through the intestinal epithelium via two transport methods: paracellular (between adjacent cells) or transcellular (through the cells) [77]. Intestinal epithelial cells can transfer NPs along the mineral elements, though their ability is limited. Transcellular transport starts with endocytosis (pinocytosis or macropinocytosis) [78]. The absorption of the NPs depends on the size, surface hydrophobicity, and electric charge [79]. The epithelium of the digestive tract is comprised of lipids, resulting in a higher absorption rate of hydrophobic NPs than hydrophilic NPs. The absorption of 100 nm NPs in the digestive tract is about 15 to 250 times higher than that of larger NPs [80]. Loeschner et al. reported that the absorption and distribution of Se from SeNPs to organs and excretion in urine showed the same results as Se (IV) used as a positive control administered through the oral route in rats. Upon administration of high dosages of SeNPs or Se (IV), high relative amounts in the liver and kidneys as compared to low dosages or controls suggested a difference in metabolism depending on dosage and form of Se [81]. In blood, Se is transported through selenoprotein P (SelP) and extracellular GPx. More than 50% of circulating Se comprises plasma selenoprotein P [82]. Se has the narrowest margin between nutritive deficiency (<40 mg/day) and toxic levels (>400 mg/day), hence the reference intake of Se in diets has been fixed in the range of 30–55 mg/day by international agencies due to linked toxic side effects [83]. However, SeNPs exhibited less toxic effects as calculated through the median lethal dose (50%, LD_50_), reduced liver impairment, and short-term side effects upon being tested in mice [84]. Qamar et al. tested dosages of 2.5 mg/kg, 5 mg/kg, 10 mg/kg, and 20 mg/kg of SeNPs and reported no significant toxic side effects oin mice spleens, livers, and kidneys and showed normal serum biochemical parameters as compared to control mice [85]. In a recent report, a comparison between SeNPs and selenomethionine (SeMet) in male C3H/HeJ mice to estimate the LD_50_ showed that SeNPs induce minor toxic effects compared to SeMet [86]. In short, SeNPs are less toxic, more bioavailable, and possess stronger biological properties than other organic and inorganic Se forms [87,88].

### 6.2. Protective Role of SeNPs against Rheumatoid Arthritis

Ren et al. reported that SeNPs dispersed in 1% phytochemical coumaric acid (CA) restored altered biochemical parameters in rheumatic rat models, suggesting notable therapeutic potential against the hallmarks of RA. The antioxidant and anti-inflammation potential of SeNPs reverted the GPx1, CAT, and COX-2 mRNA expression and restored the levels of TNF-α, IL-1β, IL-6, and MCP-1 [89]. Based on the observation of Ren et al., the mechanism of action of SeNPs in RA was further elucidated by Zhang et al. via the observing the role of SeNP supplementation on activated neutrophils with reference to neutrophil extracellular traps (NETs). OS is the central mediator that leads to the induction of NETs [90,91]. SeNPs reduced the NETs as exhibited in SeNP treatment RA mice models. The neutrophils in RA have shown increased levels of OS, lowered action of antioxidant enzymes, and more inflammation related cytokines, and treatment of SeNPs attenuated these abnormal levels as a possible mechanism of decreased NETs [92]. *Foeniculum vulgare Mill.*-derived SeNPs exhibited antioxidant and anti-inflammation potential in arthritic albino, laboratory-bred strain of the house mouse (BALB/c) mice models; SeNPs at a dosage of 10 mg/kg showed significant potential in a 2,2-Diphenyl-1-Picrylhydrazyl (DPPH)test, reduction in paw volume, and reversal in biochemical parameters in a treated BALB/c group [93]. Qamar et al. reported the potential of SeNPs prepared from *Trachyspermum ammi* against RA in BALB/c mice models. SeNPs exhibited correction in a manner independent of dose in the redox state through the upregulation of antioxidant defenses and a reduction of paw edema as compared to the diseased group [85]. Hence, the antiarthritic potential of SeNPs is perhaps due to the reduction of ROS, inflammation related markers, and increase in antioxidant protection as established from recent research.

### 6.3. Role of SeNPs against ROS and Inflammation Markers

Free radical oxygen containing molecules and their subsequent precursors produced in biological systems are called ROS and include superoxide (O_2_^−^), hydrogen peroxide (H_2_O_2_), and hydroxyl radical (OH). Moreover, reactive nitrogen species (RNS), such as NO and ONOO^−^, also possess similar properties and are hence studied to comprehend the pathophysiology of OS-related pathologies [94]. ROS and RNS have a dual role as they can be both beneficial and/or harmful for biological systems [95,96]. The formation of ROS/RNS is an unavoidable consequence of oxidative burst. OS is a state of imbalance between pro-oxidants and antioxidants favoring pro-oxidant events [97]. Excess production of ROS and RNS lead to protein oxidation and nitration, lipid peroxidation, and DNA fragmentation that eventually influences gene expression, cell membrane structures, as well as enzyme functions. O_2_^−^ is produced in vivo via NADPH oxidase, xanthine oxidase, leakage from electron transport chain of mitochondria, or activated immune cells [94]. Nitric oxide (NO) is a crucial molecule in the inflammatory and degradative cascade of arthritis. The formation of NO is elevated in inflammatory arthritis and contributes towards inflammation of synovium and cartilage [98]. Both OS and inflammation are deemed to be the most important role players in the pathogenesis of RA [99]. Unbalanced buildup of ROS/RNS leads to pathophysiological events, such as neurodegenerative disorders, cancer, cardiovascular maladies, and chronic inflammation, because of OS [100,101]. RA is also accompanied by such oxidative bursts that have a direct contribution to the proliferation or destruction of synovium [102,103]. The chief sources of OS in RA are (1) continual production of ROS due to chronic inflammation by activated leucocytes; (2) recurrent hypoxia–reperfusion phases superimposed on an ROS-rich, hypoxic milieu in the RA synovial joint; (3) amplified metabolic levels in synovium; and (4) free transition metal ions and molecules having transition metal ions released during tissue damage acting as catalysts of ROS [97]. ROS acts as a second messenger to activate NF-κB that further orchestrates the expression of a range of genes related to inflammatory response [104]. TNF-α and IL-1β are known regulators of NF-κB activation cascade and are under its transcriptional control [104,105]. Moreover, ROS is also needed for the action of other transcription factors such as activator protein 1 and hypoxia-inducible factor-1α [106]. Hypoxia-reperfusion also triggers HIF-1α and NF-κB and by doing so contributes to the maintenance of an inflammatory loop [97]. Cyclooxygenase 2 and TNF-α are the target genes that are activated through hypoxia-induced NF-κB signaling cascade [107,108]. Together, these mechanisms add up to a malicious cycle where the antioxidant defense is overwhelmed and the damaging levels of ROS result in a state of OS [97].

ROS are controlled through the actions of antioxidant defenses, including (CAT), superoxide dismutase (SOD), glutathione reductase (GR), glutathione peroxidase (GPx), and thioredoxin reductase (TrxR) [109]. Glutathione peroxidase is the first selenoenzyme to be discovered and an essential part of the antioxidant system in a living beings [110,111]. GPxs have a profound role in providing protection to cells against oxidative damage from ROS such as hydrogen peroxides, superoxides, and hydroxyl radicals [112,113]. GPx reduces H_2_O_2_ to H_2_O through glutathione (GSH) that oxidizes to glutathione disulfide (GSSG) [114]. GSH is an intracellular thiol antioxidant and lower levels of GSH lead to higher levels of ROS production and results in an imbalance in immune response and inflammation as well as an increased risk to infection [115]. The other significant group of selenoenzymes are TrxRs [116,117]. TrxRs sustain the thioredoxin/thioredoxin reductase system in a reduced state, using thioredoxin as a substrate for removal of damaging H_2_O_2_ [118]. Selenoproteins P, K, and W might also have a substantial function in providing defense against damaging ROS and RNS [14,119,120,121]. Especially selenoprotein P, which functions as an extracellular antioxidant linked to vascular endothelium that reduces ONOO− levels [122]. Besides its notable antioxidant role, Se has also been described to have potential against inflammation-induced damage [43,123,124]. GPx and selenoprotein P are also implicated in the regulation of inflammation-related responses [125]. It has been reported that GPx has a role in arachidonic acid breakdown and alters prostaglandin and leukotriene synthesis [126]. GPx degrades hydroperoxide intermediates in the cyclooxygenase and lipoxygenase pathways, reducing the production of inflaming prostaglandins and leukotrienes [127]. The endoplasmic reticulum (ER) transmembrane selenoproteins S and K are also reported to be linked to inflammation and immune regulation [128]. Selenoprotein K is especially sensitive to Se status in human peripheral leukocytes, suggesting that this protein might have a relevant function in immune cells in addition to its ER stress associated functions [129].

**Figure 2:**
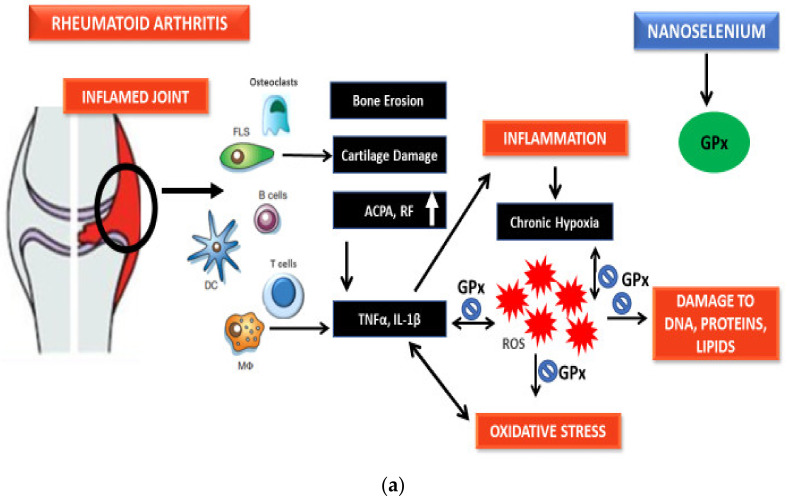

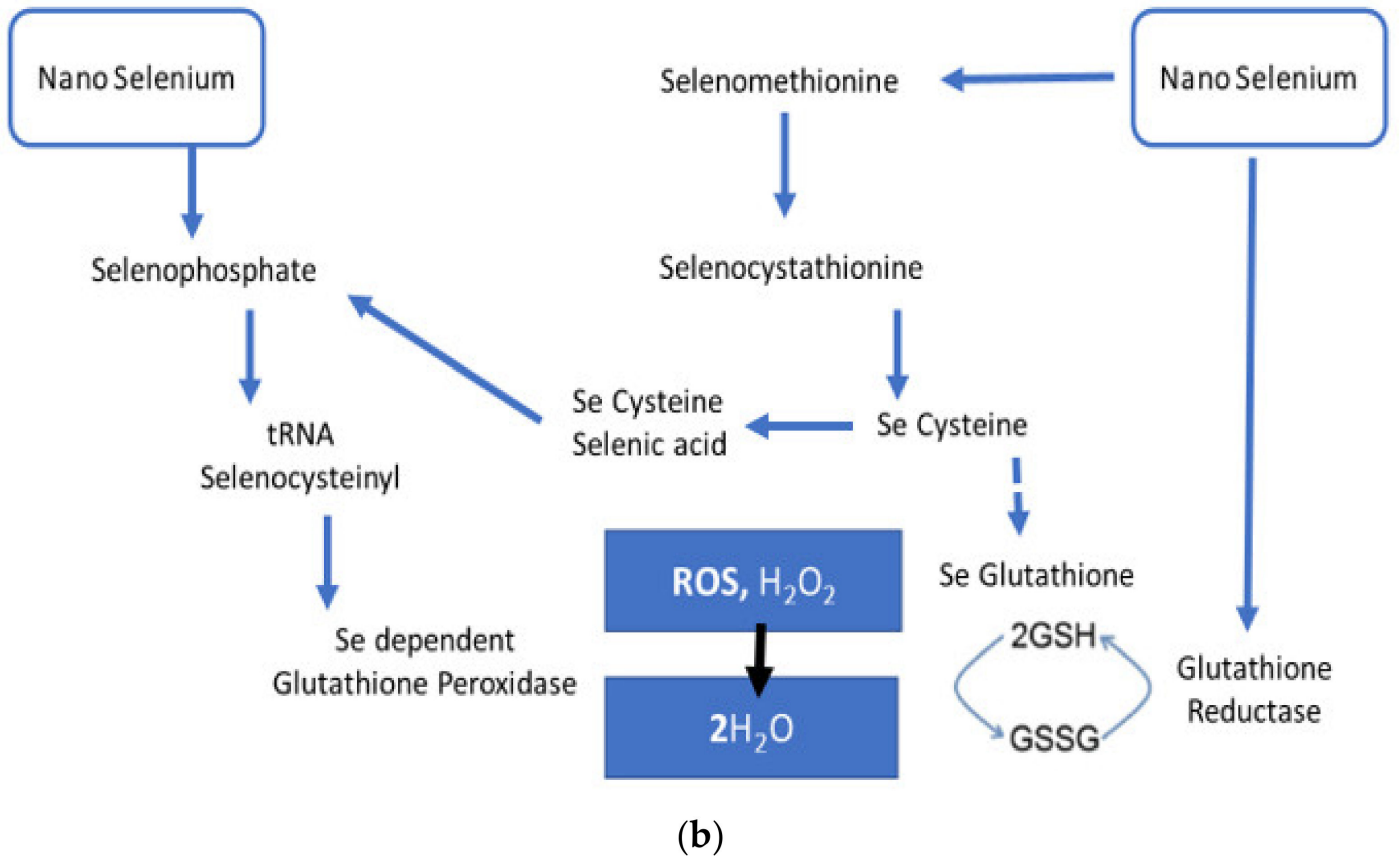
(**a**) Probable antioxidant mechanism of selenium nanoparticles. (**b**) Probable antioxidant mechanism of selenium nanoparticles.

Se has been studied at great length due to its function in the regulation of ROS/RNS and inflammation as well as its role as an antioxidant and anti-inflammatory substance. Increased levels of TNF-α and decreased levels of GPx1 alongside upregulated NF-κB have been observed in macrophages cultured in an Se-deficient environment [130,131]. Se-glutathione, along with glutathione peroxidase, plays an important role in ROS and H_2_O_2_ neutralization. In a study, it was reported that nanoselenium stimulated the expression of glutathione peroxidase, an Se-dependent enzyme, through the selenophosphate formation, which is an essential part of selenocysteine-specific tRNA [132]. Figure 2 explains the mechanism of nanoselenium in response to ROS-induced OS and inflammation in detail.

## 7. Significance of Biogenic Nanoparticles

NPs can be produced using physical, chemical, or biological methods, but using physical and chemical approaches have related drawbacks, including the need for expensive equipment, harmful chemicals, high-temperature conditions, and acidic pH, that may prove to be toxic and dangerous for further biological applications [5]. Of late, biological methods have gained much approval and remarkable interest as they offer reliable, nontoxic, energy-efficient, ecofriendly, low-cost treatments that have great potential in the pharmacological market [133,134]. The biological approach includes the usage of natural organisms, microorganism, microalgae, enzymes, and plant extracts for making NPs [135]. Green approach-based production of NPs is a favorable substitute to generate stable and biocompatible NPs for diverse medical applications [136]. Microorganisms such as bacteria and fungi as well as some plants are predominantly reported as viable for the synthesis of biological agents [137]. SeNPs have proven to be more effective than other forms of Se at increasing the expression of selenoproteins and scavenging free radicals. Recent studies of biogenic SeNPs produced using plants, bacteria, fungi, and proteins have been summarized with their role against OS and inflammation as follows.

### 7.1. Potential of Plant-Derived SeNPs

*Zingiber officinale* (ginger)-derived SeNPs have been tested against aluminum chloride-induced hepatorenal toxicity in rats and provided significant antioxidant benefits through reduction in GSH, SOD, GPx, and malondialdehyde (MDA) levels [138]. Ginger-extract-made SeNPs have also been reported to have antioxidant-mediated anti-inflammatory properties and improved nicotine-induced renal inflammation-mediated impairment in rats [139]. Menon et al. also reported the antioxidant potential of *Zingiber officinale*-derived SeNPs through DPPH tests [140]. Given that long-term treatment using DMARDs and NSAIDs leads to renal and hepatic toxic damage in rheumatoid patients, [141,142] ginger-derived SeNPs can be a possible option to diminish the treatment-associated toxic effects in rheumatoid patients. Kameswari et al. reported on the potent free radical scavenging and anti-inflammatory potential of SeNPs derived from *Acalypha indica* extract [143]. Other SeNPs derived from plants have also showed significant antioxidant potential [144,145,146,147]. Based on the data reported above, Table 2 shows the detailed aspects of the mentioned studies. The antioxidant action of these NPs may be associated with the functional groups bound to them that originate from the extract material and may have a role in the bioreduction and capping of SeNPs [140]. However, the antioxidant potential of NPs is reported to be reliant on the particle size and concentration. The comparison of antioxidant potential based on DPPH test results between studies 3, 5, and 8 in Table 2 of the same sodium selenite content reference standard shows the dependence of antioxidant activity on particle size and concentration. The SeNPs from aloe vera with a particle size of 7 to 48 nm exhibited the highest antioxidant potential. Likewise, between studies 4 and 5, SeNPs from *Diospyros montana* showed high antioxidant potential. Moreover, the increase in antioxidant potential associated with increase in concentration was also evident.

### 7.2. Potential of SeNPs from Bacteria

*Lactococcus lactis NZ9000*-derived SeNPs exhibited antioxidant and anti-inflammation effects in porcine intestinal epithelial cells (IPEC-J2) induced by H_2_O_2_ [148]. *Lactobacillus casei ATCC* 393-derived SeNPs showed antioxidant potential in H_2_O_2_-stimulated oxidative damage models of human colon mucosal epithelial cells (NCM460) and in diquat-induced intestinal barrier malfunction models in C57BL/6 mice [149,150,151]. Provided that a high risk of intestinal damage has been reported in rheumatoid patients due to possible influence of the underlying disease and long-term use of NSAIDs [152], *Lactococcus lactis NZ9000* and *Lactobacillus casei ATCC 393* SeNPs can be a suitable remedial option. *Cyanobacterial* strain-made SeNPs showed excellent antioxidant properties, among which arthrospira indica SOSA-4-prepared SeNPs provided the best score for antioxidant potential as indicated through IC50, DPPH, and SOR testing [153]. Table 3 shows results from the reported studies discussed above. The MDA and GPx activities of studies 1 and 2 with the same sodium selenite content were notable. In the cases of both strains, the derivative SeNPs exhibited lower MDA activity as compared to the H_2_O_2_ model group, but more GPx activity. However, the GPx activity of particles with 50 to 80 nm size had greater GPx activity as compared to particles with a 143 nm size. Hence, the size impact was evident.

### 7.3. Potential of SeNPs from Fungi

*Ganoderma lucidum* polysaccharide (SPS)-decorated SeNPs showed anti-inflammation effects in LPS-stimulated murine macrophages through the inhibition of NF-κB, JNK1/2, and p38 MAPKs signaling cascades [154]. *Ulva lactuca* polysaccharide (ULP)-coated SeNPs also showed anti-inflammation effects through the inhibition of NF-κB-mediated hyperinflammation in murine acute colitis models [155]. Given that ulcerative colitis (UC) and RA have a notable correlation [156], it can be proposed that ULP-SeNPs can be a potential therapeutic or combinational alternative for UC and RA remedial regimens. The results of the studies discussed above are shown in Table 4. SeNPs from both studies 1 and 2 showed a remarkable reduction in the mRNA levels of iNOS, IL-10, and TNF-α in LPS-stimulated murine macrophages and DSS-induced colitis mice models.

### 7.4. Potential of SeNPs from Proteins

Proteins have been reported to be associated with SeNPs, however the underlying functions and molecular mechanisms are still not known [157,158]. It was observed that intracellular organic matter on the proteins act as capping agents, and hence influence the surface charge and stability of the SeNPs. Proteins enriched with charged amino acids can control the formation as well as stabilization of the SeNPs [159]. This protein-derived control of the size of NPs has countless applications for industrial-scale production [157]. Keratin and bovine serum albumin have been used to prepare SeNPs and tested in vitro and in vivo for their antioxidant potential in H9c2 cell lines [160]. Furthermore, bovine serum albumin was also used in another report to prepare SeNPs and the resultant NPs were checked for their antioxidant potential through several in vitro tests and in female Swiss albino mice [161]. In another report, melatonin was used to make SeNPs and showed anti-inflammation and antioxidant potential in mice models [162]. Table 5 provides an account of the above-mentioned studies. The results of dichlorofluorescein (DCF)H-DA from the SeNPs of studies 1 and 2 showed notable antioxidant potential.

## 8. Conclusions

SeNPs have vast applications from diagnostics to treatment of otherwise nondiagnosable and untreatable health-related contradictions. Se is an important trace element that plays an essential role in bodily functions in both healthy and diseased individuals. Biogenic SeNPs have shown exceptional potential to be used as a therapeutic alternative for RA due to their unique properties such as large surface area, nano size, surface charge and chemistry, solubility, and multifunctionality. SeNPs derived from biological methods are less toxic as well as more bioavailable than other organic and inorganic forms of Se. SeNPs can act as both pro-oxidants and antioxidants based on subsequent duration, dose, frequency, as well as oxidation state. SeNPs that have potent antioxidant and anti-inflammation effects can be used not only to diminish ROS, OS, and inflammation, but also in combination with present regimens to reduce associated complications linked to available treatment options. Comparisons of different studies included in the article showed that the antioxidant and anti-inflammatory activity is dependent on the size and concentration of the SeNPs. There are toxicity and dosage concerns about the use of SeNPs in a therapeutic role, but these can be overcome via preclinical studies in animal models. Thus, preclinical studies are the need of the hour before SeNP-based treatments can see the light of the day. SeNPs present a cost-effective alternative that can be derived in an ecobeneficial manner and prove a spectacular therapeutic agent if further research is carried out finding valuable information in future. It is also of immense importance to translate these findings from bench to bedside with proper commercial regulations and market policies to innovate healthcare.

## Figures and Tables

**Table 1:** Current treatment options for RA and related side effects.

Drugs	Mode of Mechanism	Side Effects
NSAIDs	Inhibition of COXs	Cardiovascular risk, gastro-intestinal disorders, and renal malfunction
GCs	Inhibition of phospholipid release	Cardiovascular disorders, osteoporosis, insulin resistance, skin thinning, hypertension, and obesity
Conventional synthetic DMARDs	Disease altering activities	Interstitial pneumonitis, myelosuppression, hepatic cirrhosis, retinopathies, hypersensitivity, and allergic reactions
Biologic DMARDs	Inhibitors to immune mediators	Bacterial infections and high costs
Targeted synthetic DMARDs	Intracellular blockers of tyrosine kinase	Infections, headaches, hypertension, nausea, diarrhea, and high cholesterol levels

**Table 2:** Potential of plant-derived SeNPs.

No.	Source	Se Precursor	Size	Shape	Potential Role	Methods	References
1.	*Zingiber officinale*	Sodium selenite	-	Spherical	Inhibited oxidative damage due to aluminum chloride in albino rats	SOD/MDA/GSH and GPx have been measured	[138]
2.	*Zingiber officinale*	Sodium selenite pentahydrate	10–30 nm	Spherical	Reported antioxidant-mediated anti-inflammatory potential	MDA, GSH, GPX, and GST levels were determined and TNFα, COX1 and COX2 levels were also measured	[139]
3.	*Zingiber officinale*	Sodium selenite	100–150 nm	Spherical	Reported antioxidant potential	DPPH assay performed	[140]
4.	*Acalypha indica*	Sodium selenite	-	-	Reported antioxidant and anti-inflammation effect	DPPH radical scavenging and albumin denaturation checked	[143]
5.	*Emblica officinalis*	Sodium selenite	20–60 nm	Spherical	Reported antioxidant potential	DPPH and ABTS assays performed	[145]
6.	*Diospyros montana*	Selenous acid	4–16 nm	-	Reported antioxidant potential	DPPH assay performed and reducing power checked	[146]
7.	*Withania somnifera*	Selenous acid	45–90 nm	Spherical	Reported moderate antioxidant effect	DPPH assay performed	[147]
8.	*Aloe vera*	Sodium selenite	7–48 nm	Spherical	Reported antioxidant potential	ABTS, DPPH, and FRAP assays performed	[144]

**Table 3:** Potential of SeNPs from bacteria.

No.	Source	Se Precursor	Size	Shape	Potential Role	Methods	References
1.	*Lactococcus lactis* *NZ9000*	Sodium selenite	143 nm	Spherical	Reported antioxidant and anti-inflammatory effect in IPEC-J2-induced by H_2_O_2_	Levels of IL-6, IL-8, IFN-γ, and TNF-α were measured alongside MDA content, T-SOD, and GPx levels	[148]
2.	*Lactobacillus casei ATCC 393*	Sodium selenite	50–80 nm	Nanosphere	Reported antioxidant potential in H2O_2_-induced oxidative damage model NCM460	MDA and GPX levels were checked	[149]
3.	*Lactobacillus casei ATCC 393*	Sodium selenite	50–80 nm	Nanosphere	Reported antioxidant potential in diquat or H_2_O_2_ caused oxidative damage in intestinal epithelial cells	ROS levels were measured	[150]
4.	*Lactobacillus casei ATCC 393*	Sodium selenite	50–80 nm	Nanosphere	Reported antioxidant potential in diquat-induced intestinal barrier dysfunction in C57BL/6 mice	SOD, Trx, and GPx1 levels were observed	[151]
5.	*Cyanobacterial strains*	Sodium selenite	-	Spherical		DPPH, IC50, and SOR scavenging assays performed	[153]

**Table 4:** Potential of SeNPs from Fungi.

No.	Source	Se Precursor	Size	Shape	Potential Role	Methods	References
1.	*Ganoderma lucidum*	Sodium selenite	25 nm	Spherical	Reported anti-inflammatory and antioxidant potential in LPS-stimulated murine macrophages	Expression levels of iNOS, IL-1β, IL-10, and TNF-α were measured and NO production was also observed	[154]
2.	*Ulva lactuca*	Sodium selenite	58–205 nm	-	Reported anti-inflammatory and antioxidant potential in mice subjected to DSS-induced colitis, colon tissues, and LPS-stimulated cells	GSH, MDA, and GPx levels were checked; COX2, iNOS, TNF-α, IL-10, and IL-6 levels were observed	[155]

**Table 5:** Potential of protein-derived SeNPs.

No.	Source	Se Precursor	Size	Shape	Potential Role	Methods	References
1.	Keratin	Sodium selenite	100–200 nm	Spherical	Antioxidant potential against 1% ethanol-induced oxidative damage in H9C2 cell line	ROS detection measurement of conversion of DCFH-DA to fluorescent DCF using a fluorescent microscope	[160]
2.	Bovine serum albumin	Sodium selenite	500–600 nm	Spherical	Antioxidant potential against 1% ethanol-induced oxidative damage in H9C2 cell line	ROS detection measurement of conversion of DCFH-DA to fluorescent DCF using a fluorescent microscope	[160]
3.	Bovine serum albumin	Sodium selenite	5–100 nm	Spherical	Significant antioxidant potential has been reported by cyclophosphamide-induced damage in female Swiss albino mice	ROS generation was measured using DCFH-DA; level of lipid peroxides formed was measured using TBA; GSH, GSSH, GPx, SOD, and CAT levels were also measured	[161]
4.	Melatonin	Sodium selenite	-	Spherical	MT-Se treated BCG/LPS-induced hepatic injury mice models showed antioxidant and anti-inflammation action	NO, MDA, SOD, and GPx levels were measured; TNF-α, IL-1β, and splenocyte proliferation levels were also observed	[162]
